# Sudden Unexpected Death in Epilepsy

**DOI:** 10.3390/neurolint14030048

**Published:** 2022-07-18

**Authors:** Teri B. O’Neal, Sanjay Shrestha, Harsimar Singh, Ihianle Osagie, Kenechukwu Ben-Okafor, Elyse M. Cornett, Alan D. Kaye

**Affiliations:** 1 Department of Family Medicine, LSU Health Shreveport/Monroe Medical Center, Monroe, LA 71202, USA; teri.oneal@lsuhs.edu (T.B.O.); sanjay.shrestha@lsuhs.edu (S.S.); harsimar.singh@lsuhs.edu (H.S.); ihianle.osagie@lsuhs.edu (I.O.); kenechukwu.benokafor@lsuhs.edu (K.B.-O.); 2 Department of Anesthesiology, Louisiana State University Health Shreveport, Shreveport, LA 71103, USA; alan.kaye@lsuhs.edu

**Keywords:** epilepsy, sudden death, cardiac arrest, arrythmia, Takotsubo cardiomyopathy, counseling, education

## Abstract

Epilepsy is a complex neurological condition with numerous etiologies and treatment options. In a subset of these patients, sudden unexpected death can occur, and to date, there are numerous explanations as to the pathophysiological mechanisms and how to mitigate these catastrophic outcomes. Approximately 2.3 million Americans have epilepsy, and nearly 150,000 people develop the condition each year. Sudden unexpected death in epilepsy (SUDEP) accounts for 2–18% of all epilepsy-related deaths and this is equivalent to one death in 1000 person-years of diagnosed epilepsy. It is more common in young adults aged 20–45. Seizures in the past year; the absence of terminal remission in the last five years; increased seizure frequency, particularly GTCS; and nocturnal seizures are the most potent modifiable risk factors for SUDEP. Patients not receiving any antiepileptic drug therapy are at higher risk of SUDEP. Patient education on medication compliance; care plans for seizure clusters (rescue medicines); epilepsy self-management programs; and lifestyle changes to avoid seizure-triggering factors, including avoiding excessive alcohol use and sleep deprivation, should be provided by health care providers. Continued research into SUDEP will hopefully lead to effective interventions to minimize occurrences. At present, aggressive control of epilepsy and enhanced education for individuals and the public are the most effective weapons for combating SUDEP. This narrative review focuses on updated information related to SUDEP epidemiology; pathophysiology; risk factor treatment options; and finally, a discussion of important clinical studies. We seek to encourage clinicians who care for patients with epilepsy to be aggressive in controlling seizure activity and diligent in their review of risk factors and education of patients and their families about SUDEP.

## 1. Introduction

Epilepsy is a common neurological diagnosis and exists when an individual who has experienced a seizure has a brain that “demonstrates a pathologic and enduring tendency to have recurrent seizures” [1]. This diagnosis predisposes affected individuals to a higher risk of premature death when compared with the general population, and some studies estimate the difference to be a 24-fold increase [2]. With more than 2 million Americans affected by this chronic illness, the clinical implications are significant.

The causes of death (CODs) in patients with epilepsy are of various types and include epilepsy-related and nonepilepsy-related entities. Nonepilepsy-related causes may include accidental deaths from drowning, burns, and motor vehicle collisions. These are external CODs and occur commonly in patients with epilepsy, and they are not part of the disease itself but may occur as a result of seizure activity. Epilepsy-related causes include status epilepticus and sudden unexpected death in epilepsy (SUDEP) [2]. This review, therefore, focuses on SUDEP as an unanticipated and devastating COD in patients with epilepsy. 

SUDEP was first reported in the medical literature by Bacon in *The Lancet* in 1868, but more than 100 years lapsed before SUDEP received an official definition [3]. In 1997, Nashef specifically identified this event as “sudden, unexpected, witnessed or unwitnessed, non-traumatic, and non drowning death in patients with epilepsy” [4]. Postmortem findings reveal no definite etiology for the patient’s demise [4]. There are several features in epilepsy that have been implicated as risk factors for SUDEP, but the definitive etiology for SUDEP is still a point of debate and research. Pathophysiological possibilities include lethal cardiac arrhythmias, autonomic dysfunction, and respiratory compromise [5]. The known risk factors include age between 20 and 40, male gender, longer duration of epilepsy, and higher frequency of generalized tonic-clonic (GTC) seizures. 

SUDEP is arguably the most feared complication of epilepsy and is responsible for about one death in 1000 person-years of diagnosed epilepsy [6]. Three thousand victims annually in the United States may seem a relatively small cohort, but the resulting public health burden is enormous. The victims of SUDEP are generally much younger than those who die from many other chronic neurological conditions. This accounts for many years of potential life lost with every individual who succumbs to SUDEP. This number is generally a better measure of the true public health burden than mortality alone [7]. 

Clinicians who care for patients with epilepsy must be aware of the potential complications of the disease, including SUDEP. Management of the disease to control GTC seizures and to mitigate adverse events is key. Better disease control is associated with a decreased likelihood of associated sudden death [8]. Increased education and awareness for patients, families, and the public could also diminish the occurrence of SUDEP [4]. This article highlights the epidemiology and suspected pathophysiology and treatment and monitoring options for minimizing the possibility of SUDEP. In summary, SUDEP is an extremely devastating cause of premature epilepsy-associated death among children and adult patient populations with epilepsy.

This narrative review focuses on updated information related to SUDEP epidemiology; pathophysiology; risk factors; treatment options; and finally, a discussion of important clinical studies. We searched the following databases: PubMed, Medline, SciHub, Cochrane Database of Systematic Reviews, and Google Scholar. We used the following MeSH terms: epilepsy/complications; death, sudden/epidemiology; death, sudden/etiology; epilepsy/mortality; arrhythmias, cardiac; Takotsubo cardiomyopathy; patient education; and humans. We tried to include as many recent manuscripts as possible (within the last three years) and included papers that were older than three years if they were particularly relevant to our topic. We also attempted to search for, use, and cite primary manuscripts whenever possible. 

## 2. Epidemiology

As one of the most common, chronic, and debilitating neurological conditions, epilepsy is a major problem today. Approximately 2.3 million Americans have epilepsy, and nearly 150,000 people develop the condition each year. Almost half a million children (from 0 to 17 years old) have epilepsy in the United States [4]. 

**Figure 1:**
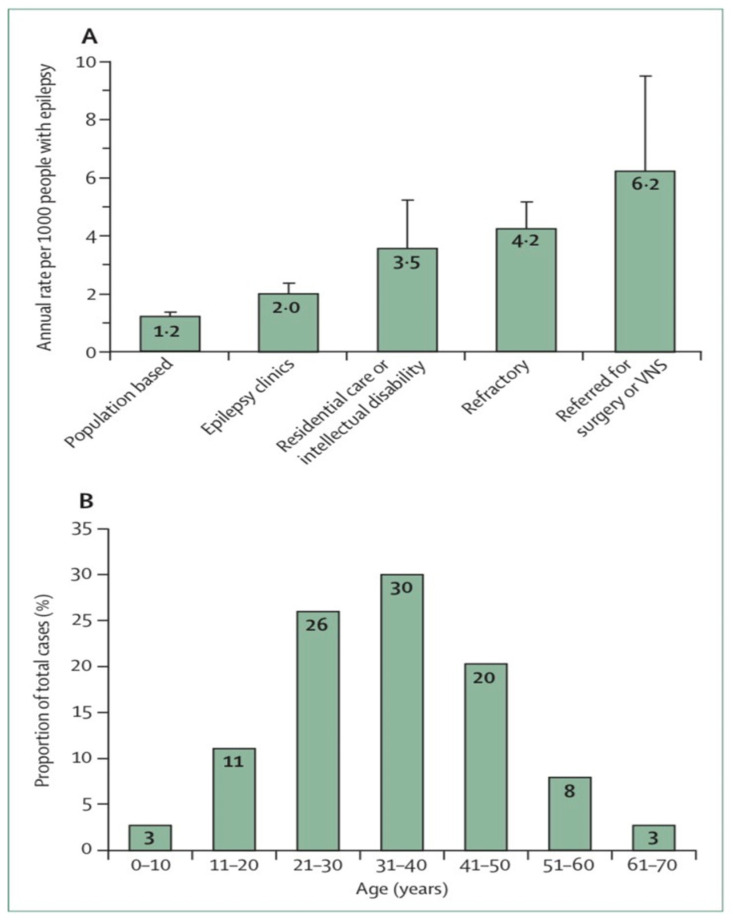
Sudden unexpected death in epilepsy epidemiology [7,14] (**A**) Estimated annual sudden unexpected death in epilepsy incidence in different epilepsy patient populations. (**B**) Distribution of sudden unexpected death in epilepsy cases by age. Error bars reflect 95% Cis. VNS = vagus nerve stimulation.

The incidence of SUDEP varies greatly depending on the population studied, how epilepsy is recorded and how epilepsy is recognized among the population [9]. A study of the number of people with epilepsy per 1000 per year related to SUDEP found from 6.3 to 9.3 risks for epilepsy surgery candidates, from 1.1 to 5.6 risks for epilepsy clinic populations with a high proportion of refractory epilepsy patients, and from 0.35 to 2.3 risks for community residents with epilepsy (Figure 1A) [10]. SUDEP is estimated to be a lifetime risk of 7 percent overall or 12 percent for those with persistent epilepsy by age 40 [11]. It has been estimated that the cumulative lifetime risk is 35 percent [9].

Sudden unexpected death in epilepsy (SUDEP) accounts for 2–18% of all epilepsy-related deaths [12]. SUDEP is estimated to occur in approximately 1.2/1000 person-years (PY) [13]. The incidence of SUDEP is low among young children, more prevalent among adolescents, highest in young adults and significantly decreased thereafter (Figure 1B) [14]. In this regard, the estimated incidence of SUDEP in children seems to be significantly lower (0.22/1000 PY) than in adults, but recent studies in children (>12 years) indicated a similar incidence to that of adults. 

### 2.1. Risk Factors

**Figure 2:**
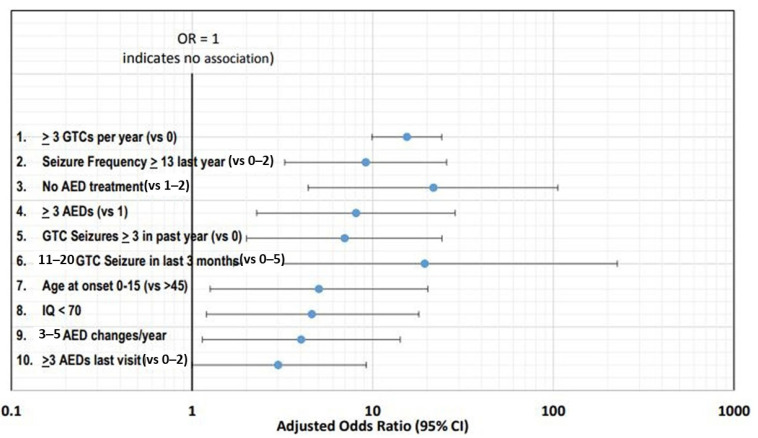
Top ten risk factors using adjusted ORs with corresponding 95% confidence intervals [16].

Understanding the risk factors for SUDEP is important for clinicians when identifying the patients at high risk. To identify top risk factors associated with SUDEP, DeGiorgio et al. studied the risk factors in studies published in core clinical journals from 1987 to 2017 [15]. In this study, crude and adjusted odds ratios or relative risk ratios (ORs or RRs) were analyzed and the risk factors were ranked using log OR/RR. Figure 2 shows the top ten risk factors using adjusted ORs with corresponding 95% confidence intervals.

The frequency of seizures has been consistently identified as the leading risk factor, with the top two risk factors being three or more GTC seizures per year and thirteen or more seizures (any type) in the past year [17,18]. 

Antiseizure medications (ASM) treatment (previously known as antiepileptic drugs or AEDs) has also been extensively investigated with SUDEP. Patients not receiving any ASM therapy are at higher risk of SUDEP [19]. Polytherapy is another leading risk factor, with three or more ASMs associated with increased risk [18]. However, it is more likely that the risk associated with polytherapy is related to severe drug resistance and high seizure frequency rather than the number of ASMs [20]. Another risk factor is frequent changes in the ASM dose [18].

All these risk factors help clinicians identify the interventions that can help reduce the risk of SUDEP. Clinicians should strive to control GTC seizures aggressively and to make their patients seizure-free whenever possible.

### 2.2. Pathophysiology

Any process that affects or diminishes the blood supply to the heart and brain can be potentially fatal. Numerous hypotheses have been put forward to explain SUDEP, including cardiac, respiratory, cerebral, and autonomic dysfunctions. 

Many genetic mutations due to their effects on cardiac, respiratory, and nervous system can potentially cause SUDEP. The Table 1 lists the genes associated with SUDEP.

#### 2.2.1. Cardiac Hypothesis

Sudden cardiac arrest has been postulated as one of the mechanisms of SUDEP, and various studies and investigations have been performed to identify specific ECG findings associated with it. Transient prolongation of the QT interval during the peri-ictal state can lead to abnormal cardiac repolarization causing ventricular tachyarrhythmia [21,22,23]. Studies have also detected a shortening of the QT interval during seizures [23,24]. It is of particular interest in people with genetically determined short QT syndrome who have high chances of reentry tachycardia [25]. Another finding is ventricular late potentials associated with seizures, which can cause ventricular tachyarrhythmias [26].

The electrophysiological changes implicated in SUDEP have been linked with many ion channel abnormalities. The mutated SCN1A gene linked with Dravet syndrome is one of the most common gene mutations associated with SUDEP [27,28]. Other mutations include genes associated with long QT syndrome or those involved with controlling cardiac rhythm, such as KCNQ1, LQTS, KCNH2, SCN5A, and ryanodine calcium channels [28,29,30,31,32]. Many of these are expressed in the heart and brain, suggesting that their mutations can predispose an individual to seizure and cardiac arrhythmia [33,34,35].

Contrary to tachyarrhythmias associated with seizures, bradyarrhythmia and asystole have also been described [36]. Ictal bradyarrhythmia is caused by seizure-like activity in both temporal lobes, leading to either deactivation of the sympathetic system or activation of the parasympathetic system [37,38,39]. It is not clear whether this can lead to sudden cardiac death, but a cardiac pacemaker has been advised to manage these patients [40,41].

In some patients with epilepsy, stress-induced Takotsubo cardiomyopathy has been reported, especially after convulsive seizures or status epilepticus. It can impair myocardial contractility, causing cardiogenic shock, cardiac arrhythmias, and SUDEP [42,43].

#### 2.2.2. Autonomic Dysfunction

It has been established that heart rate increases during seizures due to autonomic system involvement [44]. Autonomic system derangements associated with epilepsy include excessive sympathetic activity, low parasympathetic activity, high vasomotor tone, and severe dysautonomia [16,45]. An important risk factor for sudden cardiac death in intractable epilepsy and antiepileptic usage is decreased heart rate variability [46]. This decrease in heart rate variability is directly related to the duration of seizure disorder, use of antiepileptic drugs, and multiple drug therapy [47,48,49,50].

#### 2.2.3. Respiratory and Cerebral Dysfunctions

Peri-ictal hypoxemia has been reported in up to 25 percent of patients with SUDEP [23]. It can result from central apnea, airway obstruction, or pulmonary edema [51,52,53,54]. The risk is increased with longer seizure activity, temporal lobe involvement, or contralateral lobe involvement [55,56].

EEG findings suggest that cardiac and respiratory dysfunction are caused by disruptions in the autonomic networks during pathological cerebral activity. A retrospective study of EEG and ECG findings in monitored cardiac arrests revealed central apnea and bradycardia following a generalized tonic-clonic seizure after initial variation of heart and respiratory rates, showing altered cardiorespiratory function leading to SUDEP [5]. Another case–control study suggested a relation between prolonged postictal generalized EEG suppression (PGES) and SUDEP [57]. Although the association between PGES and SUDEP could not be replicated in a subsequent study, still, these studies were able to demonstrate that PGES follows convulsive seizures [58].

**Figure 3:**
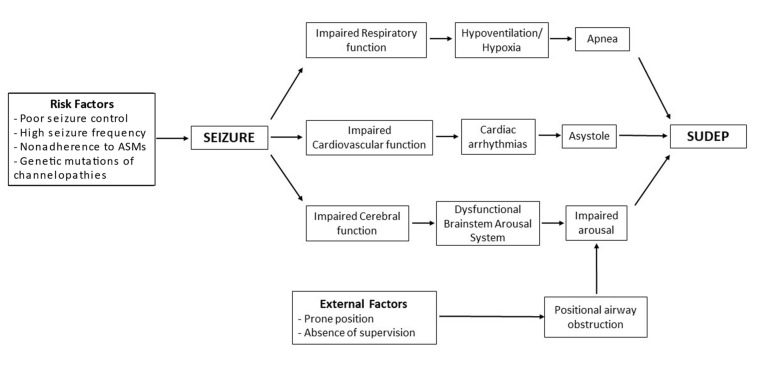
Pathophysiology model of SUDEP.

Figure 3 is an attempt to show the interactions between different mechanisms and risk factors that can potentially lead to SUDEP.

### 2.3. Treatment for the Prevention of Sudden Unexpected Death in Epilepsy

SUDEP is a devastating cause of premature epilepsy-associated death among children and adult patient populations with epilepsy. The prevention of this enigma is still an arduous task, as the exact pathophysiology of SUDEP is unknown. Measures aimed at reducing the risk of SUDEP largely target postulated theories on its pathophysiology and a few potentially modifiable risk factors in affected patients. These risk factors have been identified from case–control, cohort studies, and systematic reviews. Some of the proposed interventions are highlighted below.

#### 2.3.1. Counseling and Education

Several societies, including the American Academy of Neurology (AAN), strongly recommend discussing tailored information about SUDEP with patients and their relatives. However, there is no evidence that the disclosure of SUDEP risk to patients is associated with improvements in medication compliance, changes in anxiety levels, or protection from SUDEP [59]. Some experts have opined that it fosters a “truth-telling relationship” between doctors and their patients while helping to define treatment goals.

Additionally, patient education on medication compliance; care plans for seizure clusters (rescue medicines); epilepsy self-management programs; and lifestyle changes to avoid seizure-triggering factors, including avoiding excessive alcohol use and sleep deprivation, should be provided by health care providers [60].

#### 2.3.2. Optimize Treatment of Drug-Resistant Epilepsy

SUDEP prevention is an important aspect of achieving seizure control with effective epilepsy treatment [61]. The risk of SUDEP is greater in patients with a higher burden of GTCS (generalized tonic-clonic seizures) [62]. Hence, adjunctive anti-seizure medications and referral for surgical evaluation of lesional epilepsy to reduce the frequency of GTCS is protective.

#### 2.3.3. Seizure-Monitoring Devices

A variety of seizure detection devices (bed sensors, alarms, and heart rate monitors) are currently available for detecting cardiorespiratory distress, thus prompting early intervention when necessary. Their use should be individualized based on a patient’s seizure profile, as evidence to support their effectiveness in preventing SUDEP is lacking. More clinical studies are required to ascertain their role in the SUDEP prevention algorithm [59,61].

#### 2.3.4. Preventing Airway Obstruction

Safety (ventilated foam) pillows and nocturnal supervision have been hypothesized to reduce the impact of post-ictal airway compromise, especially when sleeping in the prone position. There are no data to support the role of safety pillows, but there is limited low-quality evidence that corroborates the benefit of nocturnal supervision in preventing SUDEP. Hence, timely supervision and prompt administration of emergency treatment post-seizure recovery are advised, especially in patients with frequent nocturnal seizures [59,61].

#### 2.3.5. Reducing Brain and Brainstem Depression from Endogenous Opioids and Adenosine

The termination of seizures is typically preceded by a surge in endogenous opioids and adenosine, which can lead to postictal apnea when in excess. Medications, including naloxone and caffeine, have been used in a few studies to attenuate the effect of endogenous opioids and adenosine, respectively. However, due to the lack of data on this intervention, there is no compelling evidence to support their benefit or use in preventing SUDEP [60].

#### 2.3.6. Cardiac and Diaphragmatic Pacing

Standard cardiac and phrenic nerve pacing holds the potential to prevent cardiorespiratory failure. This needs to be explored further as prevention modalities in the SUDEP algorithm.

#### 2.3.7. Clinical Practice Recommendations

The clinical practice recommendations are presented in Table 2.

## 3. Recent Clinical Findings

Much of the current understanding of SUDEP has come from studies published in the last twenty years. The National Library of Medicine (PubMed) identified more than 1300 articles published after the year 2000 instead of just 109 articles published between 1970 and 2000. Recent findings are only beginning to throw light on the prediction, prevention, and treatment of SUDEP. These findings in terms of clinical findings, efficacy, and comparison studies are summarized in Table 3 and Table 4.

The exact pathophysiology of SUDEP remains unclear. Surges et al. concluded that there was likely a complex interplay of factors, including dysregulation of cardiac and respiratory function [70]. Gunbey et al. also demonstrated impaired cardiac autonomic response to sympathetic stimulation in a small cohort of patients who died from SUDEP [71]. Decoding the cardiac, autonomic, respiratory, and polygenic contributors to SUDEP would answer many questions about prevention and prediction. At the time of writing this manuscript, there is no validated biomarker or predictor of SUDEP risk. This has not been from a lack of effort. A 2021 prospective, multicenter epilepsy monitoring study investigated autonomic and breathing biomarkers in patients ≥18 years with intractable epilepsy and generalized convulsive seizures [67]. Post-convulsive central apnea occurred in 22% of seizures, and the authors concluded it could be a SUDEP biomarker.

A 2021 retrospective case–control study evaluated the utility of the SUDEP-7 inventory [72]. The SUDEP-7 inventory is one of the tools used in practice and clinical research to estimate SUDEP risk. The authors reported that when compared with matched controls, mean SUDEP-7 scores were significantly higher in persons who died from SUDEP. This provided the first external validation of a risk prevention model for SUDEP. The authors also proposed a novel tool—the SUDEP-3—which improved the predictive performance. Furthermore, several epidemiologic studies provide data showing the potential for certain autonomic indices to predict the risk of SUDEP. Heart Rate Variability (HRV), an inexpensive and easily available clinical tool, has shown promise. Altered HRV is common in patients with epilepsy, particularly temporal lobe epilepsy and drug-resistant seizures [68]. A 2018 retrospective case–control study evaluated the utility of HRV as a predictor for SUDEP risk in individuals with epilepsy due to mutations in sodium channel (SCN) genes [68]. The authors concluded a direct association between interictal HRV and SUDEP in epilepsy due to SCN mutations. More evidence for the utility of HRV as a biomarker for SUDEP was reported in 2021 by Sivathamboo et al. [67]. A retrospective nested case–control study compared HRV in SUDEP cases and living epilepsy controls. Five-minute interictal ECG recordings were evaluated in 31 SUDEP cases and 56 controls who had been admitted for video EEG monitoring. The study found that reduced short-term low-frequency power (a measure of HRV) was associated with SUDEP. More work will be essential to determine the implications for clinical management.

Sadly, incidence rates of SUDEP have shown a correlation with socioeconomic status (SES). One study published in 2020 reported significantly higher incidence rates in people with the lowest SES than those with the highest SES [73]. This could be due to reduced access to specialty care and availability of AED among people with epilepsy with lower SES.

The use of DNA sequencing has improved the understanding of the genetic associations of SUDEP. The data suggest that a sizeable portion of SUDEP cases demonstrate pathogenic mutations or prospective pathogenic variants in cardiac arrythmia and epilepsy genes [74]. In a 2019 systematic review by Chahal et al. that examined the genetics of patients diagnosed with SUDEP or any seizure disorder with a history of sudden unexplained death, the most common variants reported were in the genes encoding sodium and potassium ion channel subunits [59]. This is highlighted in Dravet syndrome (DS), a treatment-resistant form of epilepsy with a high risk for SUDEP, where loss-of-function mutations have been reported in the SCN1A gene, which encodes a subtype of voltage-gated sodium channel in the brain. The authors also concluded that most SUDEP events were oligogenic or polygenic. Expanding the list of SUDEP candidate genes will be crucial in managing risk for patients and their families.

The incidence rate of SUDEP in people with Dravet syndrome (DS) is ~6× higher than in people with other forms of epilepsy [65]. A 2021 pooled analysis of three double-blind, placebo-controlled trials evaluated the impact of fenfluramine (FFA) on expected SUDEP mortality rate in patients with Dravet syndrome. A total of 732 patients diagnosed with DS and for whom seizures were uncontrolled with the current anti-seizure medication regimen were randomized to receive either fenfluramine or placebo in addition to the current ASM regimen. Fenfluramine treatment reduced the incidence rates of SUDEP and all-cause mortality to 1.7 deaths per 1000 person years. This was significantly lower than the all-cause mortality and SUDEP rates of 15.84 and 9.32 deaths per 1000 person years reported by Cooper et al. in patients with DS receiving the standard of care. The authors concluded that DS patients treated with FFA experienced much lower SUDEP-related and all-cause mortality rates compared with a historical natural history cohort. Though exciting, this analysis raises further questions for research. For instance, are these findings applicable to SUDEP unrelated to DS? The authors recommended further studies to evaluate the sustainability of the observed effects and the pharmacology of FFA.

Neuromodulation devices have shown promise in the treatment of drug-resistant epilepsy. Currently, all three FDA-approved devices—vagal nerve stimulation (VNS), responsive neurostimulation (RNS), and deep brain stimulation of the anterior nucleus of the thalamus (ANT DBS)—have shown a reduction in SUDEP incidence [62]. A 2021 multicenter, double-blind, randomized, sham-controlled trial evaluated the long-term efficacy and safety of ANT DBS in a patient who had been followed for at least seven years and some for ten years [64]. The study reported a SUDEP rate of 2 deaths per 1000 person-years, which is below the reported rate in patients with drug-resistant epilepsy (6.3–9.3 deaths per 1000 person-years) [8]. See Table 3 and Table 4.

## 4. Conclusions

Epilepsy affects more than 2 million individuals in the United States alone and millions more worldwide. It is one of the neurological conditions most often encountered by primary care clinicians worldwide, outnumbering stroke and second only to headache [4]. There are multiple CODs in epilepsy patients, but undoubtedly, the most devastating is SUDEP. The most common nontraumatic COD in epilepsy, SUDEP, is responsible for as many as 18% of all deaths in epilepsy patients and one death per 1000 person-years of diagnosed epilepsy [6]. About 3000 deaths per year in the United States are attributed to SUDEP. Still, most of these individuals are significantly younger than those who die from other causes such as Alzheimer’s disease and stroke. The number of person-years lost to SUDEP represents a significant impact on the public health burden and provides a better measure than mortality numbers alone [7]. The devastating effect of SUDEP on families and populations makes it a topic of concern for any clinician who cares for patients with epilepsy.

The pathophysiology responsible for SUDEP is a point of ongoing debate and investigation. The formal definition of “sudden, unexpected, witnessed or unwitnessed, non-traumatic, and non drowning death in patients with epilepsy” excludes external causes [4]. Research is now targeting the systems that seem most likely to be triggered to malfunction by seizure activity, namely the cardiac, respiratory, and autonomic nervous system [4].

Risk factors have been identified with ongoing research. Known risk factors for SUDEP included male gender, age in the third and fourth decades of life, and higher frequency of GTC seizures. The frequency of GTC seizures is consistently identified as a leading risk factor [56,57]. Information regarding the risk for SUDEP allows clinicians to assess individual patients and to determine when changes in therapy or initiation of additional monitoring are appropriate. A variety of seizure detection devices has become available to detect deterioration in cardiorespiratory status, thereby allowing for immediate intervention in epilepsy patients. At present, evidence is lacking for their effectiveness, and additional study is required to determine where these potential interventions have a role in SUDEP prevention [63]. The optimization of seizure control is protective by reducing the frequency of GCT seizures, thereby lessening the risk of SUDEP in epilepsy patients [61,62]. This should be the primary focus for clinicians.

Education and counseling also have a very important role in managing patients with epilepsy and their families. Providing clear information about the risk of SUDEP has not been proven to improve compliance or outcomes; however, there may be significant benefits to these discussions [4,59]. Most caregivers who lost a loved one to SUDEP express regret at not knowing this possible outcome before the terminal event s [4]. Another benefit to the enhanced communication regarding SUDEP is heightened public awareness of this as a complication of epilepsy. Much like sudden infant death syndrome (SIDS), which was addressed in the form of public education such as the Back to Sleep campaign, increased SUDEP awareness might improve the overall incidence of this devastating event [4].

SUDEP is a catastrophic event, both for patients and families and public health burden worldwide. Continued research into the etiology of this entity will hopefully lead to additional effective interventions to minimize occurrences. In the meantime, aggressive control of epilepsy and enhanced education for individuals and the public are the most effective weapons to combat SUDEP [4,61]. The authors hope this review will encourage clinicians who care for patients with epilepsy embrace these treatment goals.

## Figures and Tables

**Table 1:** Genetic association with SUDEP.

Gene	Clinical Condition	Function	SUDEP Hypothesis
SCN1A	Dravet syndrome, generalizedepilepsy with febrile seizures	Sodium channel	Increases epilepsyseverity by postictal parasympathetichyperactivity
SCN2A	Epileptic encephalopathy	Sodium channel	Increases severity of epilepsy
SCN8A	Epileptic encephalopathy	Sodium channel	Increases severity of epilepsy
PRRT2	Benign familial infantile seizures	Proline-rich transmembrane protein 2	Potential interaction withSNAP-25 and presynapticneurotransmitter release
DEPDC5	Focal epilepsy	G-protein signaling pathway, inhibits the mTORC1 pathway	Potential increase in severity of epilepsy
CSTB	Unverricht-Lundborg disease	Inhibits intracellular thiol protease, prevents protease leakage from Lysosomes	Increases severity of epilepsy and neurological impairment due toprogressive myoclonic epilepsy
TSC1, TSC2	Tuberous sclerosis complex	Downregulates mTORC1 pathway	Potential increase in severity of epilepsy
HCN2	Generalized epilepsy	Contributes to spontaneous rhythmic activity in SA node and brain	Potential impairment inbrainstem or cardiac pacemaker cells
KCNQ1	Long QT syndrome	Potassium channel; ventricular repolarization	Potentialarrhythmogenic effect
KCNH2	Long QT syndrome	Potassium channel;repolarization of cardiac action potential	Uncertain
SCN5A	Long QT syndrome	Sodium channel; rapid depolarizing sodium current underlying cardiac action potential upstroke	Potential combination of epilepsy and arrhythmia
NOS1AP	Long QT syndrome	Cytosolic protein that binds to neuronal nitric oxide synthase	Potential combination of epilepsy and arrhythmia
RYR2	Sudden cardiac death	Cardiac ryanodine receptor 2; intracellular calcium release channel, coupling excitation–contraction	Potential combination of epilepsy and arrhythmia
HCN4	Bradycardia, sick sinus syndrome	Potassiumchannel; slow kinetics of activation and inactivation, cardiacpacemaker role	Variant identified in SUDEP

**Table 2:** Clinical practice recommendations for SUDEP treatment.

Grade B	Effective epilepsy treatment to decrease the burden of GTCS protects against SUDEP. Providers should use appropriate anti-seizure medications and combine ASM where necessary to achieve seizure control, while actively involving patients in their care and weighing the safety profile of the medications.
Grade C	Based on risk profile and psychosocial circumstances, clinicians should selectively counsel patients with frequent uncontrolled nocturnal seizures on nocturnal supervision, as this is protective against SUDEP.
Grade C	Prompt referral for surgical evaluation of drug-resistant epilepsy/lesional epilepsy is of paramount importance in reducing the risk of SUDEP.

**Table 3:** Clinical efficacy and safety.

Author (Year)	Intervention	Results and Findings	Conclusion
J. Helen Cross et al. (2021) [63]	Fenfluramine (FFA) added to anti-seizure medication for Dravet syndrome patients to assess its effect on the SUDEP mortality rate.	All-cause and SUDEP mortality rates were significantly lower than expected compared to estimates from literature studies.	FFA might have a role in reducing SUDEP in patients with Dravet syndrome. More studies will be required to ascertain if this effect is sustainable and applicable to other causes of SUDEP.
V. Salanova et al. (2021) [64]	Adults with severe epilepsy underwent deep brain stimulation surgery with leads implanted in the anterior thalamus (ANT DBS). They were followed up over 7–10 years.	The observed SUDEP rate was lower for patients with drug-resistant epilepsy, including patients being treated with adjunctive ASMs or considered for epilepsy surgery.	ANT DBS is associated with sustained improvement in seizure reduction over time. This reduction is possibly responsible for the reduction in SUDEP risk.
Vilella et al. (2019) [65]	Patients with intractable epilepsy (*n* = 87) underwent monitoring of autonomic and breathing biomarkers in epilepsy monitoring units.	Post-convulsive central apnea (PCCA) was associated with near-SUDEP phenomena and SUDEP.	The authors suggest PCCA is a possible SUDEP biomarker.

**Table 4:** Comparison studies.

Author (Year)	Groups Studied	Results and Findings	Conclusion
Myers et al. (2018) [66]	HRV data were compared between a group of patients with SCN mutation drug-resistant epilepsy and a control group of non-SCN drug-resistant epilepsy.	SUDEP patients had more severe autonomic dysregulation. This dysregulation was worse in the SCN mutation group.	The authors suggest autonomic dysfunction is associated with SUDEP risk in patients with epilepsy due to SCN mutations.
Sivathamboo et al. (2021) [67]	A retrospective nested case–control study evaluated interictal ECG recordings among patients admitted for video EEG recording	Normalized LFP was lower in SUDEP cases than in matched controls. Every 1% reduction in normalized LFP conferred a 2.7% increase in latency to SUDEP.	The authors conclude reduced short-term LFP is associated with SUDEP. They suggest that increased LFP may be associated with longer survival.
Cihan et al. (2020) [68]	Over 2 years and in 3 diverse geographic regions, SUDEP rates plus certain demographic, biometric, and clinical variables were compared with community SES.	159 SUDEP cases in the lowest quartile zip codes and 49 cases in the highest quartile zip codes. No reported difference in age, sex, BMI, epilepsy etiology, circumstances of death, nonadherence to medication, and comorbid conditions between highest and lowest quartile SES zip codes.	The authors concluded SUDEP rates were >2 times higher among people with epilepsy living in lowest income communities compared to the highest income communities.
Rasekhi et al. (2021) [69]	A retrospective case–control study evaluated 48 patients with epilepsy who underwent EEG monitoring and subsequently died of definite or probable SUDEP. Two matched controls with epilepsy were identified for each individual who died of SUDEP.	SUDEP-7 scores were significantly higher in the SUDEP group than in matched controls, both at the time of admission and last follow-up.	The authors conclude that results support the ability of SUDEP-7 inventory to predict SUDEP. However, it does not enhance the prediction of SUDEP over-generalized tonic-clonic seizure or seizure frequency alone. They propose a new tool—SUDEP-3 inventory, which improves predictive performance.

## Data Availability

The data are available from the authors upon request.
